# m^6^A-mediated upregulation of LINC01003 regulates cell migration by targeting the CAV1/FAK signaling pathway in glioma

**DOI:** 10.1186/s13062-023-00386-6

**Published:** 2023-06-03

**Authors:** Xiaolong Zhu, Xingwei Wu, Hui Yang, Qiancheng Xu, Mengying Zhang, Xiaocen Liu, Kun Lv

**Affiliations:** 1 Central Laboratory, The First Affiliated Hospital of Wannan Medical College (Yijishan Hospital of Wannan Medical College), Wuhu, 241001 People’s Republic of China; 2 Key Laboratory of Non-Coding RNA Transformation Research of Anhui Higher Education Institutes (Wannan Medical College), Wuhu, 241001 People’s Republic of China; 3 Non-Coding RNA Research Center of Wannan Medical College, Wuhu, 241001 People’s Republic of China; 4 Anhui Provincial Clinical Research Center for Critical Respiratory Disease, Wuhu, 241001 People’s Republic of China; 5 Department of Critical Care Medicine, The First Affiliated Hospital of Wannan Medical College (Yijishan Hospital), Wuhu, 241001 People’s Republic of China; 6 Department of Nuclear Medicine, The First Affiliated Hospital of Wannan Medical College (Yijishan Hospital of Wannan Medical College), Wuhu, 241001 Anhui People’s Republic of China

**Keywords:** METTL3, LINC01003, Migration, FAK, Glioma

## Abstract

**Background:**

Long non-coding RNAs (lncRNAs) play important roles in the progression of glioma. Here, we examined the potential functions of a lncRNA, LINC01003, in glioma and characterized the underlying molecular mechanisms.

**Methods:**

The GEIPA2 and Chinese Glioma Genome Atlas (CCGA) databases were employed to analyze gene expression and the overall survival curve in patients with glioma. The functions of LINC01003 in glioma growth and migration were assessed by loss-of-function experiments in vitro and in vivo. RNA sequencing was used to determine the signaling pathways effected by LINC01003. Bioinformatics analysis and RNA immunoprecipitation (RIP) assays were used to explore the mechanism underlying the *N*6-methyladenine (m^6^A) modification-dependent upregulation of LINC01003 in glioma.

**Results:**

LINC01003 expression was upregulated in glioma cell lines and tissues. Higher LINC01003 expression predicted shorter overall survival time in glioma patients. Functionally, LINC01003 knockdown inhibited the cell cycle and cell proliferation and migration in glioma cells. Mechanistically, RNA sequencing revealed that LINC01003 mediated the focal adhesion signaling pathway. Furthermore, LINC01003 upregulation is induced by m^6^A modification regulated by METTL3.

**Conclusion:**

This study characterized LINC01003 as a lncRNA that contributes to tumorigenesis in glioma and demonstrated that the LINC01003-CAV1-FAK axis serves as a potential therapeutic target for glioma.

**Supplementary Information:**

The online version contains supplementary material available at 10.1186/s13062-023-00386-6.

## Introduction

Glioma is one of the most common primary tumors in human central nervous system (CNS), with high rates of occurrence, invasiveness, and recurrence. The overall survival time is short, and the 5-year mortality rate is high [1]. According to the 2021 WHO Classification of Tumors of the Central Nervous System, gliomas are mainly divided into astrocytoma, oligodendrogliomas, glioblastoma, angiocentric glioma, and astroblastoma [2]. The 2022 Cancer Statistics of China indicate that glioma is in the top 10 cancer types by incidence and mortality [3]. Although the treatment strategies for glioma patients include surgery, radiotherapy, chemotherapy, and immunotherapy, the prognosis of glioma patients does not improve significantly upon treatment [4]. Therefore, it is necessary to understand the mechanism underlying the progression of glioma in order to determine clinical biomarkers for timely intervention.

Long non-coding RNAs (lncRNAs) are a group of non-coding RNAs that contain more than 200 nucleotides. Accumulating evidence in recent years has suggested that lncRNAs are essential for diverse cellular and molecular mechanisms [5]. LncRNAs serve as competing endogenous RNAs (ceRNAs) that bind to mircroRNAs (miRNAs) and regulate the expression of mRNAs. LncRNAs also function as scaffolds or guides to modulate protein–protein or protein-DNA interactions [6]. Therefore, lncRNAs are multifunctional regulators associated with DNA replication, transcription, and gene expression. There are several studies showing that lncRNAs are abnormally expressed in glioma and affect multiple cellular processes, including the cell proliferation, migration, apoptosis, and the cell cycle [7, 8]. To date, a large number of glioma-related lncRNAs have been identified. Nevertheless, the molecular mechanism underlying the involvement of lncRNAs in the progression and metastasis of glioma has yet to be clarified.

*N*6-methyladenine (m^6^A) RNA modification is one of the most abundant epigenetic processes in eukaryotic mRNA [9, 10]. m^6^A is also found in lncRNAs and serves an important role in the progression of glioma [11, 12]. m^6^A is often enriched in stop codons, 3′-untranslated regions (UTRs), and splicing sites and plays a regulatory role in almost every step of the lncRNA life cycle, including transcription, splicing, nuclear export, localization, stability, and degradation [13].

In this study, we demonstrated that lncRNA LINC01003 is highly expressed in glioma and is associated with the poor clinical prognosis of glioma patients. We confirmed that LINC01003 promotes glioma proliferation and migration through in vitro and in vivo functional assays. Furthermore, we showed that LINC01003 can modulate an CAV1/focal adhesion kinase (FAK) signaling pathway in glioma. The RNA m^6^A methyltransferase METTL3 was shown to epigenetically mediate the upregulation of LINC01003 in glioma.

## Methods

### Clinical specimens and databases

Glioma tissues (n = 66) and normal brain tissues (n = 14) were obtained from patients who had undergone surgery in the Department of Neurosurgery of the First Affiliated Hospital of Wannan Medical College. The fresh tissue samples were immediately preserved in liquid nitrogen and stored at − 80 °C until RNA was extracted. This project was reviewed and approved by the Ethics Committee of the First Affiliated Hospital of Wannan Medical College. All participants gave written informed consent to allow their tissue to be used for scientific research. The transcriptional data and overall survival in glioma were downloaded from GEPIA2 (Gene Expression Profiling Interactive Analysis 2, http://gepia2.cancer-pku.cn/) [14] and the CGGA database (the Chinese Glioma Genome Atlas, http://www.cgga.org.cn/) [15].

### Cell lines and culture

The human glioma cell lines (U87MG, U251, U373, and T98G) and normal human astrocyte cell line (HEB) were obtained from American Type Culture Collection (ATCC, Manassas, USA) and cultured in DMEM (Hyclone, GE Healthcare Life Sciences, USA) medium with 10% fetal bovine serum (FBS; Gibco, Life Technologies, USA). All cell lines were grown in a humidified incubator at 37 °C containing 5% CO_2_.

### Transfection with siRNA

The lncRNA smart silencer negative control (NC) siRNA, LINC01003 smart silencer siRNA, negative control (NC) siRNA, and *METTL3* siRNA were purchased from RiboBio (Guangzhou, China). The sequence of the siRNA targeting *METTL3* was CAAGTATGTTCACTATGAA. A 6-well plate was seeded with glioma cells at a density of 15 × 10^4^ cells per well. Twenty-four hours later, the smart silencer and siRNA (100 nM) were transfected in cells using the riboFECT™ CP Transfection Kit (RiboBio) following the manufacturer's instructions.

### Infection with lentivirus

Recombinant lentiviruses for knocking down LINC01003 (sh-LINC01003) and the corresponding control viruses (sh-NC) were purchased from HANBIO (Shanghai, China). Glioma cell U87MG (15 × 10^4^ cells/well) were seeded into a 6-well plate and transfected with the lentivirus according to the manufacturer’s instructions. Infected cells were treated with 5 μg/ml puromycin for more than 7 days.

### Real-time qPCR (RT-qPCR) analysis

Total RNA from tissue or cell lines was isolated using TRIzol reagent (Ambion, Life Technologies, USA). The RNA was then reverse transcribed to complementary DNAs (cDNA) by RevertAid First Strand cDNA Synthesis Kit (Thermo Scientific, Lithuania, USA). RT-qPCR was performed using a QuantiNova™ SYBR^®^ Green PCR Kit (Qiagen, Hilden, Germany) with a Bio-Rad CXF96 PCR system (Hercules, CA, USA) according to the manufacturer's instructions. GAPDH was used as reference gene for mRNA and lncRNA detection. Relative mRNA and lncRNA expression was determined by the 2^−ΔΔCt^ method. The qRT-PCR primers were purchased from Sangon Biotech (Shanghai, China). LINC01003: F-5′-TCTCTGCGTCGCTCCTTCTCGC-3′, R-5′-CCTTCCGCGCCAAGTAGTGC-3′; CAV1: F-5′-GACTCGGAGGGACATCT CTACACC-3, R-5′-GTCGTACACTTGCTTCTCGCTCAG-3′.

### Cell proliferation and cycle assay

EdU staining assay (Cell-LightTM Apollo 488 Stain Kit, RiboBio) was used to detect cell proliferation following the manufacturer’s protocol. Briefly, transfected cells were seeded in 96-well plates and incubated with DMEM medium containing EdU for 2 h. Following cell fixation (4% formaldehyde) and cell membrane permeation (0.5% Triton X-100), the cells were stained with Apollo dye solution and Hoechst33342. The images were photographed with a Nikon Ti-U fluorescent microscope (Tokyo, Japan). The EdU-positive cells were counted using Photoshop CS6 (Adobe Systems Incorporated, San Jose, CA, USA).

For the cell cycle assay, transfected cells were collected and fixed with 75% pre-chilled ethanol for 24 h at 4 °C. Then the cells were stained with RNase A/PI (propidium iodide) mixture and incubated in the dark at 37 °C for 30 min. The cells were detected by a CytoFLEX flow cytometer (Beckman Coulter, CA, USA). The cell cycle was analyzed with Modfit LT32 software (Verity Software House, Topsham, ME).

### Cell migration assay

Cell migration was determined by a transwell assay using a transwell chamber with an 8.0-mm pore size (Corning, NY, USA). Then 2 × 10^4^ transfected cells, which were suspended in 100 μl of serum-free BMDM medium, were seeded in the upper chamber and 600 μl of BMDM medium containing 10% FBS was added to the lower chamber. Then the cells were incubated for 24 h, and the bottom side of the upper chamber was fixed with 4% formaldehyde for 10 min. After staining with 0.1% crystal violet, the cells were captured by an inverted microscope (Nikon Ti-U). The migrated cells were counted using Photoshop CS6 (Adobe Systems Incorporated).

The migration ability of the cells was also determined by a wound-healing assay. The transfected cells were cultured to 100% confluence in a 6-well plate and scratched with a 200-μl pipette tip. After washing with PBS, the cells were incubated in DMEM medium without FBS. The wound closures were captured using an inverted microscope (Nikon Ti-U) at h 0, 24, and 48.

### Western blots

The protein expression of p-FAK, FAK, E-Cadherin, N-Cadherin (Rabbit mAb; CST), matrix metallopeptidase 2 (MMP2), MMP9, and CAV1 (Rabbit Polyclonal; Proteintech) was determined as described previously [16, 17]. The total protein of cells was extracted using Laemmli 2 × concentrate sample buffer (Sigma, MO, USA). The proteins were separated by 10% polyacrylamide gel electrophoresis and transferred to 0.22-nm nitrocellulose membranes (Pall Corporation, East Hills, USA). Then the membranes were blocked with 5% BSA (bovine serum albumin) and incubated with primary and secondary antibodies. After incubation with an enhanced chemiluminescence kit (ECL, Millipore, Burlington, USA), the protein bands were captured by a ChemiDoc Touch Imaging System (Bio-Rad, Hercules, USA).

### RNA sequencing (RNA-seq) analysis

Glioma cells were transfected with lncRNA smart silencer NC (n = 2) or LINC01003 smart silencer (n = 2) siRNA for 48 h. The RNA-seq analysis was performed by Novogene Co., Ltd (Beijing, China). RNA integrity was determined by the RNA Nano 6000 Assay Kit using the Bioanalyzer 2100 system (Agilent Technologies, CA, USA). The clustering of the index-coded samples was conducted by TruSeq PE Cluster Kit v3-cBot-HS (Illumia) on a cBot Cluster Generation System. Following cluster generation, the library was sequenced on the Illumina Novaseq platform (Illumina, CA, USA). Genes with a∣log2FoldChange∣ ≥ 0 and adjusted *P* value ≤ 0.05 between NC and LINC01003 smart silencer cells were assigned as significantly differentially expressed genes (DEGs). The Cluster Profiler R package was used to analyze the statistical enrichment of DEGs in KEGG pathways.

### In vivo* xenograft model*

BALB/c nude mice (5 weeks old) were acquired from the Experimental Animal Center of Qinglongshan (Nanjing, China) and housed in a specific pathogen-free (SPF) mouse colony. For in vivo tumorigenesis experiments, U87MG cells (1 × 10^7^) were suspended in 100 μl PBS and implanted subcutaneously into the mice. The width (W) and length (L) of tumors were monitored every 5 days with a caliper. The tumor volume was calculated using the following formula: W^2^ × L × 0.5. After four weeks, the mice were sacrificed, and the tumors were isolated and weighed. The experiments using the nude mice were approved by the ethics committee of the First Affiliated Hospital of Wannan Medical College.

### ***m***^***6***^***A modification prediction***

The online tool RMBase v2.0 (http://rna.sysu.edu.cn/rmbase/) [18], which integrates epitranscriptome sequencing data, was used to explore the m^6^A modification of LINC01003. The m6A2Target (http://m6a2target.canceromics.org) database was used to predict the relationship between m^6^A enzymes with LINC01003 expression.

### RNA immunoprecipitation

The m^6^A-modified LINC01003 levels were determined using the Magna RIP RNA-binding Protein Immunoprecipitation Kit (Millipore) combined with RT-qPCR. The RIP assay for RNA binding with anti-m^6^A (Mouse monoclonal, Abcam, MA, USA) or anti-IgG (CST) was carried out in accordance with the manufacturer’s instructions. Then RT-qPCR was performed to detect the expression level of LINC01003 m^6^A modification.

### Statistical analyses

All experimental data are presented as the mean ± SD from three independent replicates. All statistical analyses were conducted using GraphPad Prism 5 (La Jolla, CA, USA). Student’s *t*-test was used to compare the data differences between two groups. One-way ANOVA followed by post hoc test was used to compare the data differences between three groups. *P < 0.05, **P < 0.01, and ***P < 0.001.

## Results

### LncRNA LINC01003 is highly expressed in glioma patients and cells

**Fig. 1:**
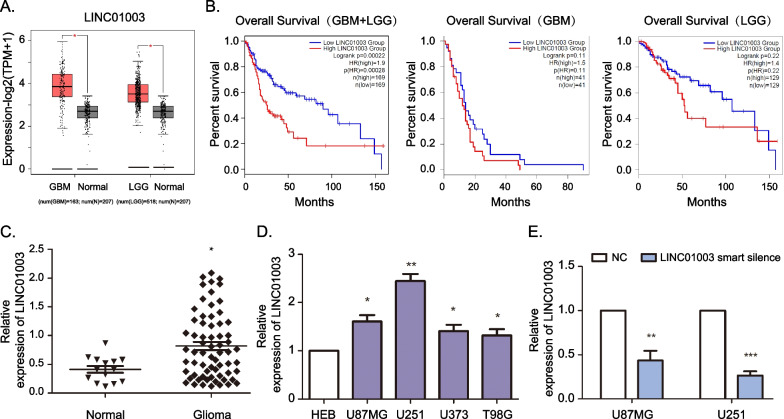
LINC01003 is upregulated in glioma tissues and cells. **A** The expression of LINC01003 in glioblastoma multiforme (GBM), lower grade glioma (LGG), and normal brain tissues from the GEPIA2 database. **B** Kaplan–Meier curves from the GEPIA2 database determined the relevance between LINC01003 expression and overall survival in GBM and/or LGG patients. **C** RT-qPCR was used to detect the relative expression of LINC01003 in 14 normal brain tissues and 66 glioma tissues. **D** RT-qPCR was used to detect the relative expression of LINC01003 in HEB, U87MG, U251, U373, and T98G cell lines. **E** Real-time qPCR (RT-qPCR) was used to detect the relative expression of LINC01003 in U87MG and U251 cells lines, where the cells were transfected with lncRNA smart silencer negative control (NC) and LINC01003 smart silencer siRNA; *P < 0.05, **P < 0.01, and ***P < 0.001

Previous research has shown that LncRNA LINC01003 expression in glioblastoma stem-like cells is significantly higher than in conventional glioma cells [5]. From this report we speculated that LINC01003 would play a critical role in glioma tumorigenesis. The GEPIA2 database showed that LINC01003 expression in GBM (glioblastoma multiforme) or LGG (lower grade glioma) is significantly higher than in normal brain tissues (Fig. 1A). Importantly, the Kaplan–Meier survival analysis from the GEPIA2 database indicated that GBM and LGG patients with a higher LINC01003 expression level were associated with shorter overall survival than those with lower LINC01003 expression level (Fig. 1B). We then performed RT-qPCR to analyze the expression of LINC01003 in brain tissues from 66 glioma patients and 14 normal patients of Yijishan Hospital. We observed that LINC01003 expression was significantly upregulated in glioma patients compared with normal controls (Fig. 1C). In addition, LINC01003 expression in glioma cell lines was significantly higher than in normal HEB cells (Fig. 1D). As LINC01003 expression was highest in U87MG and U251 cells, LINC01003 smart silence was subsequently transfected into U87MG and U251 cells. RT-qPCR assays showed that LINC01003 smart silence system significantly knockdown LINC01003 expression (Fig. 1E). Taken together, these results suggested that LINC01003 expression was upregulated in glioma and positively associated with poor prognosis.

### Effects of LINC01003 on glioma cell cycle and cell proliferation

**Fig. 2:**
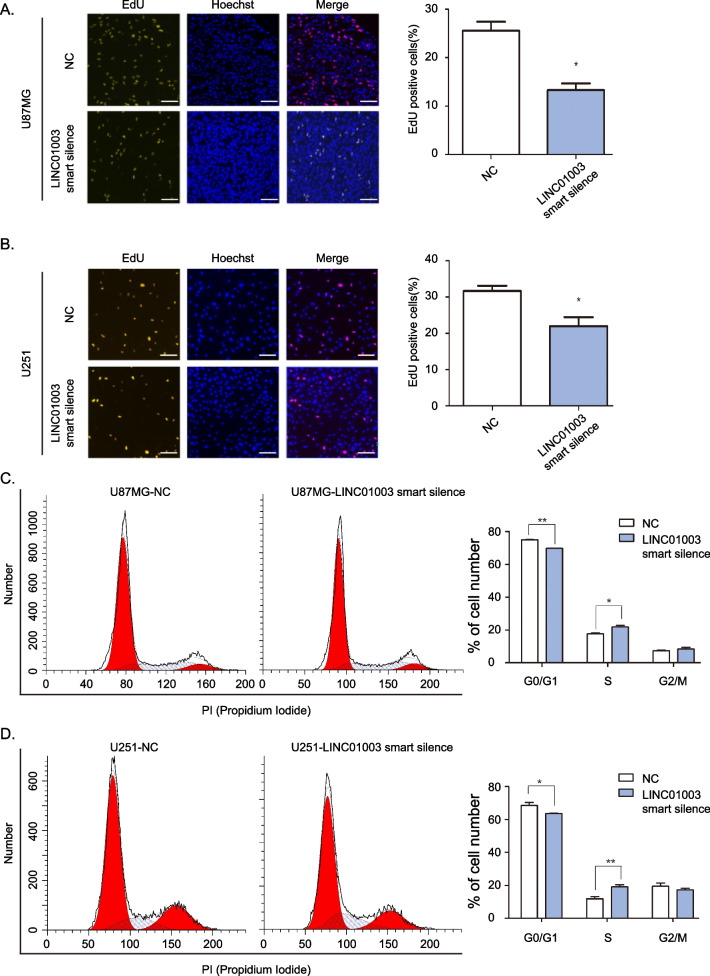
Effects of LINC01003 on the proliferation and cell cycle of glioma cells. **A**–**B** EdU (5-Ethynyl-2′-deoxyuridine) staining assay was performed to determine the proliferation of U87MG and U251 cells with LINC01003 knockdown. The percentage of EdU-positive cells were counted; scale bar, 200 μm. **C**–**D** Flow cytometry with propidium iodide (PI) staining was used to detect the cell cycle. A histogram showing the proportion of glioma cells in G0/G1, S, and G2/M phase. Data represent the mean ± SD from three independent experiments; *P < 0.05 and **P < 0.01

To further verify the function of LINC01003 in glioma, we determined the effect of LINC01003 on cell proliferation and the cell cycle by silencing LINC01003 in U87MG and U251 glioma cells using lncRNA smart silence. EdU assays indicated that LINC01003 knockdown significantly inhibited DNA replication in glioma cells (Fig. 2A–B). Uncontrolled cell cycle progression is considered to be a key marker to promote malignant proliferation of tumor cells. Thus, inhibition of cell cycle is an effective strategy in the clinical treatment of tumors. We then employed flow cytometry assays to determine the effect of LINC01003 on the cell cycle. We found that LINC01003-silenced glioma cells showed changes in the distribution of the cell cycle, with increased arrest at S phase and decreased proportion of G0/G1 phase (Fig. 2C–D). These results suggested that LINC01003 positively mediates glioma cell proliferation in vitro.

### Effects of LINC01003 on glioma cell migration

**Fig. 3:**
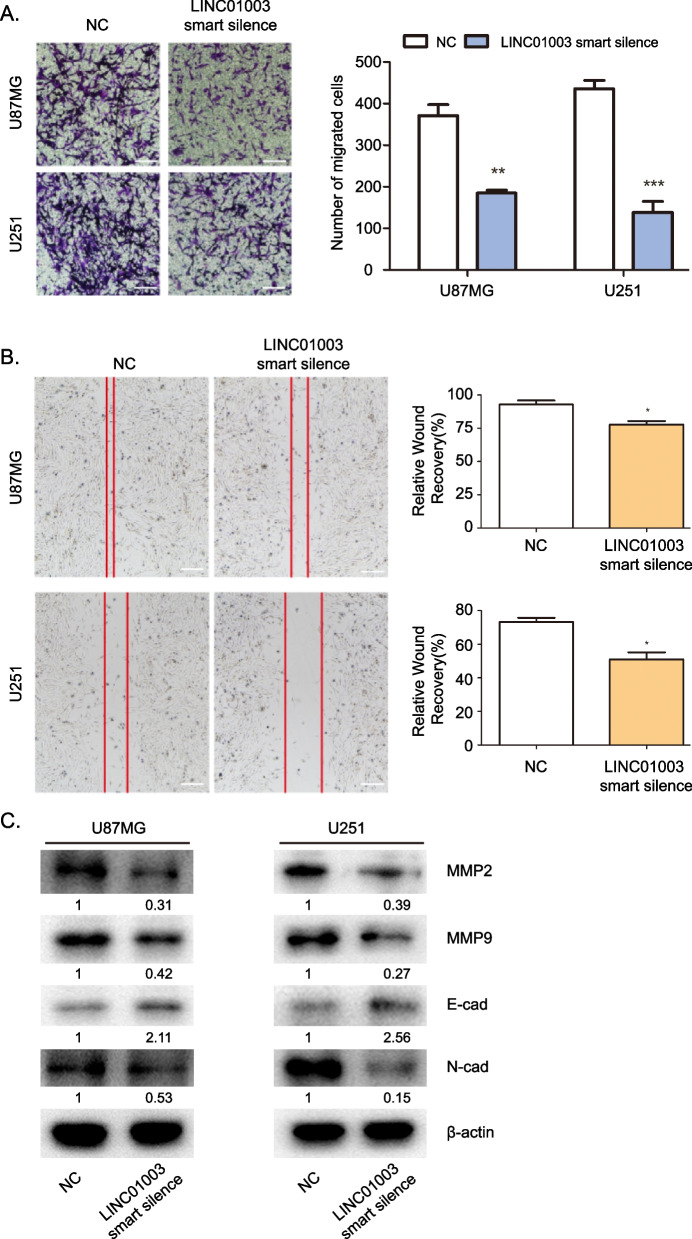
Effects of LINC01003 on the cell migration of glioma cells. **A** A transwell assay was employed to determine the migration of U87MG and U251 cells with LINC01003 knockdown. The glioma cells that migrated were counted; scale bars, 200 μm. **B** Wound healing assays was also employed to determine the migration of U87MG and U251 cells with LINC01003 knockdown. The scratch was measured 0 and 48 h after the scratch was performed; scale bars, 200 μm. **C** Western blot was used to determine the expression of matrix metallopeptidase 2 (MMP2), MMP9, E-Cadherin (E-cad) and N-Cadherin (N-cad) in U87MG and U251 cells with LINC01003 knockdown. *P < 0.05, **P < 0.01, and ***P < 0.001

Next, we examined the effects of LINC01003 on the migration of glioma cells by transwell and wound-healing assays. In the transwell assays, LINC01003 knockdown decreased the number of migrated cells (Fig. 3A). Wound-healing assays revealed that LINC01003 depletion decreased the speed of cell migration (Fig. 3B). In order to verify that LINC01003 knockdown reduced cell migration, we detected the protein expression of key regulators required for cell migration. MMP2 and MMP9 are two matrix metalloproteinases that play a critical role in tumor cell invasion and metastasis [19, 20]. Epithelial–mesenchymal transition (EMT), which is involved in the early stage of metastasis, is characteristic by N-cadherin (N-Cad) upregulation and E-cadherin (E-Cad) downregulation [21, 22]. LINC01003 knockdown significantly inhibited the expression of MMP2 and MMP9, whereas LINC01003 silencing increased the expression of E-Cad and decreased the expression of N-Cad (Fig. 3C). Collectively, this result indicated that LINC01003 knockdown inhibits the migration of glioma cells.

### LINC01003 regulates the focal adhesion kinase (FAK) signaling pathway

**Fig. 4:**
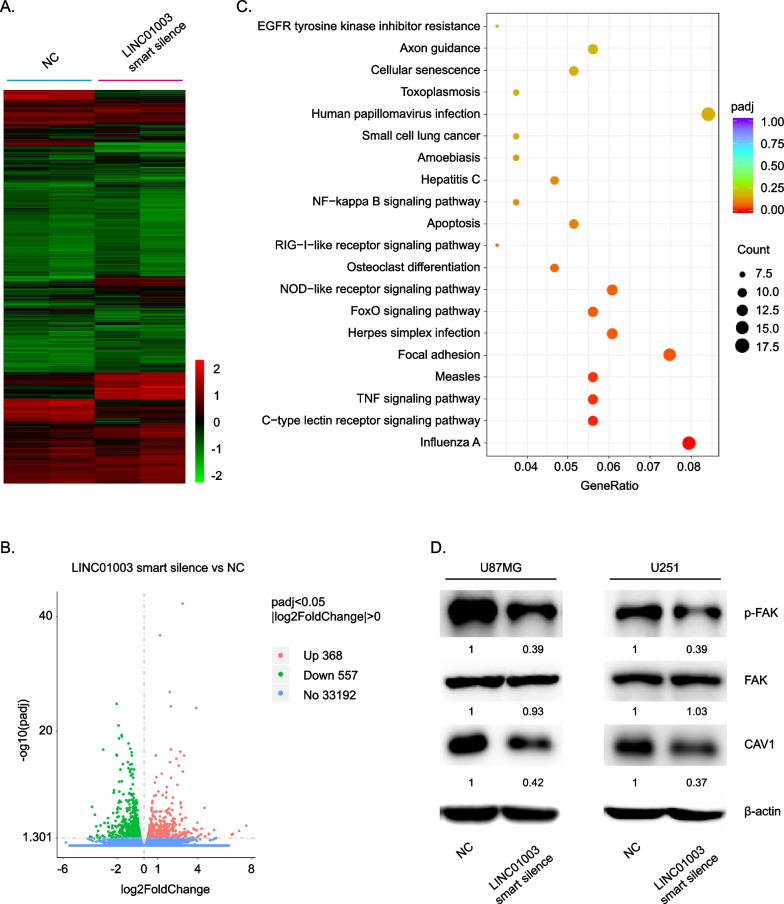
Knockdown of LINC01003 inhibited the CAV1-FAK signaling pathway. **A** The heatmap reflected the differently expressed genes (DEGs) regulated by LINC01003 knockdown. **B** A volcano plot of DEGs from (**A**). **C** Kyoto Encyclopedia of Genes and Genomes (KEGG) pathway analysis was performed for the 557 downregulated genes regulated by LINC01003 knockdown from (**B**). **D** Western blot was used to detect the expression levels of p-FAK and CAV1 in U87MG and U251 cells with LINC01003 knockdown. Up: upregulation; Down: downregulation; No: no change

To investigate the mechanism of LINC01003 action in glioma, RNA transcriptome sequencing was performed in U251 glioma cells with or without LINC01003 knockdown. LINC01003 suppression upregulated 368 genes and downregulated 557 genes (∣log2FoldChange∣ ≥ 0, adjusted *P* value ≤ 0.05) (Fig. 4A–B and Additional file 1: Table S1). KEGG pathway enrichment analysis revealed that the 557 downregulated genes were enriched in influenza A, c-type lectin receptor signaling pathway, TNF signaling pathway, measles, focal adhesion, herpes simplex infection, and FoxO signaling pathway (Fig. 4C and Additional file 2: Table S2). Focal adhesion ranked fifth in the KEGG enrichment category and included 12 genes (PIK3R3, PGF, VCL, LAMB3, THBS2, CAV1, KDR, PRKCA, LAMC2, MYL5, TNC, LAMA2, PIK3R1, BIRC3, ITGB6, and MYLPF). Our sequencing data suggested that LINC01003 mediates the expression of focal adhesion-related genes, indicating that LINC01003 might regulate focal adhesion in glioma cells. Focal adhesion kinase (FAK) is a key component of focal adhesions and crucial for cancer cell migration [23–25]. We found that LINC01003 depletion significantly reduced FAK phosphorylation in glioma cells (Fig. 4D).

CAV1 (Caveolin-1), a membrane protein of caveolae, was reported to be involved in invasion via the FAK-mediated adhesion signaling pathway [26]. The GEPIA2 database showed that CAV1 expression in GBM or LGG was significantly higher than in normal brain tissues (Additional file 3: Fig. S1A). The CGGA database showed that CAV1 expression was increased in glioma tissues with advanced tumor stage (Additional file 3: Fig. S1B). The GEPIA2 and CGGA databases demonstrated that glioma patients with higher CAV1 expression level were associated with shorter overall survival than those with a lower CAV1 expression level (Additional file 3: Fig. S1C and D). Next, western blot and RT-qPCR were performed to verify whether LINC01003 could regulate CAV1 expression. Consistent with the transcriptome sequencing data, CAV1 expression was decreased in the LINC01003 knockdown cells (Fig. 4D and Additional file 3: Fig. S1E). Collectively, these data demonstrated that LINC01003 may regulate FAK phosphorylation through CAV1.

### *LINC01003 knockdown inhibited glioma proliferation *in vivo

**Fig. 5:**
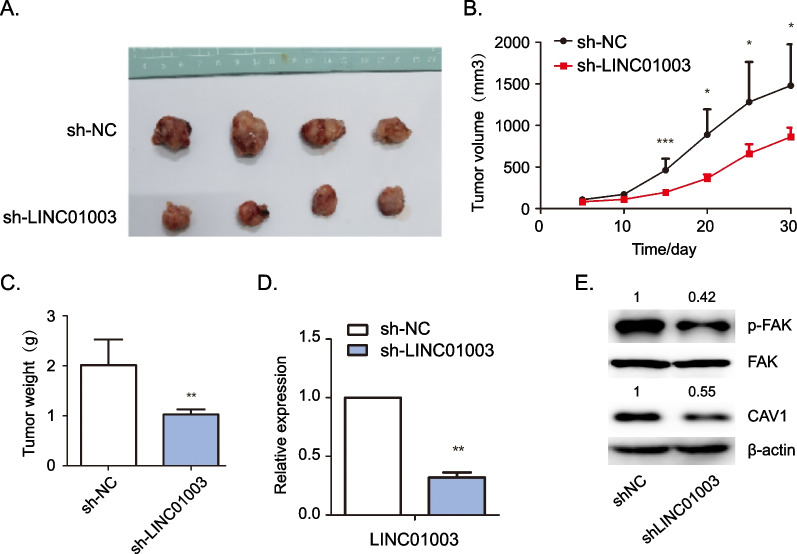
LINC01003 knockdown represses glioma growth in vivo. **A** A subcutaneous xenograft tumor model was generated by transfecting U87MG cells with shNC or shLINC01003. Tumors were removed from the nude mice and photographed at the end of experiment. **B** Once the mice were injected with the cells, he tumor volume in nude mice was measured every 5 days. The volume curves were plotted from day 5 to day 30. **C** Quantification of tumor weight in nude mice from (**A**). **D** RT-qPCR was used to determine the expression of LINC01003 in the tumors from nude mice. **E** Western blot was used to detect the expression levels of p-FAK and CAV1 in the tumors from nude mice. Data represent the mean ± SD; *P < 0.05, **P < 0.01, and ***P < 0.001

We further employed a xenograft tumor model to assess the tumor-suppressive effects of LINC01003 in vivo. The tumor growth in nude mice injected with sh-LINC01003 cells was slower as compared with those injected with sh-NC cells (Fig. 5A). LINC01003 knockdown inhibited glioma tumor volume and weight in nude mice (Fig. 5B–C). RT-qPCR assays showed that LINC01003 expression was decreased in the sh-LINC01003 xenograft tumors (Fig. 5D). The expression of CAV1 and p-FAK was then examined by western blot in xenograft tumors. As shown in Fig. 5E, the expression of CAV1 and p-FAK was significantly downregulated in the LINC01003-silenced group. In conclusion, these results suggested that knockdown of LINC01003 inhibits glioma growth in vivo.

To determine the role of the LINC01003/CAV1 axis in glioma, we employed functional rescue experiments in vitro and in vivo. Genetic addition of CAV1 alleviated the inhibitory effect of LINC01003 deficiency on the migration and p-FAK levels of glioma cells (Additional file 4: Fig. S2A and B). The xenografts result in nude mice further indicated that overexpression of CAV1 partially relieved the inhibition of tumor formation induced by knockdown of LINC01003 in vivo (Additional file 4: Fig. S2C). These findings suggested that the LINC01003/CAV1 axis regulates glioma tumorigenesis in vitro and in vivo.

### ***m***^***6***^***A modification is associated with LINC01003 expression in glioma cells***

**Fig. 6:**
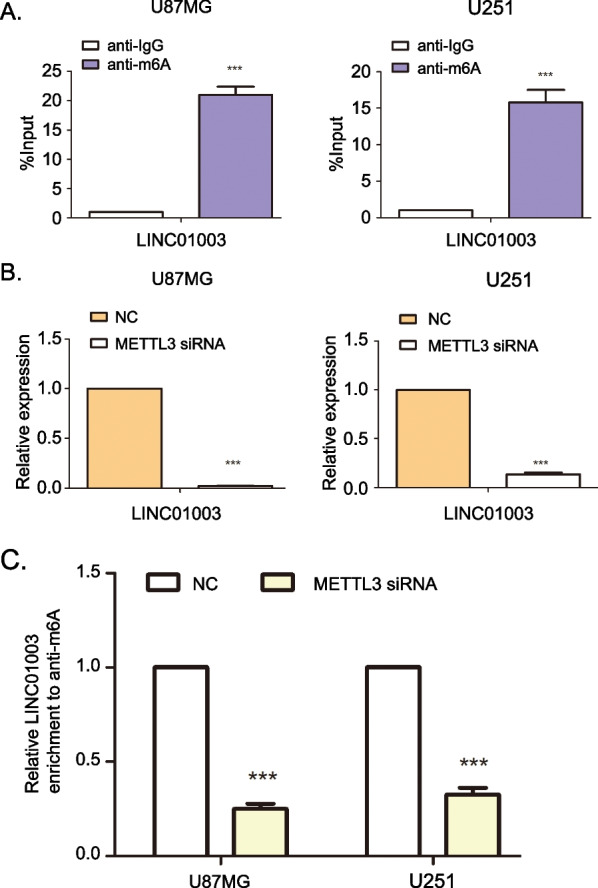
*N*6-methyladenosine (m^6^A) modification regulates LINC01003 expression in glioma cells. **A** A RNA immunoprecipitation (RIP) assay combined with RT-qPCR was used to determine the relative m^6^A enrichment at LINC01003 in U87MG and U251 cells. **B** RT-qPCR was used to determine the expression of LINC01003 in glioma cells with NC or METTL3 siRNA. **C** A RIP assay combined with RT-qPCR was used to determine the relative m^6^A enrichment at LINC01003 in glioma cells with NC or METTL3 siRNA; ***P < 0.001

Recent advances have suggested that *N*6-methyladenine (m^6^A) modifications play a critical role in the regulation of the posttranscriptional level of lncRNAs. Thus, we speculated that m^6^A modifications were responsible for the upregulation of LINC01003 in glioma. The m^6^A modification analysis online software, RMBase v2.0 database, predicted 15 potential m^6^A modification sites in the exon of LINC01003 transcript (Additional file 5: Table S3). Then the m^6^A modification level of LINC01003 in glioma cells was determined by RNA immunoprecipitation and RT-qPCR assays. Compared with the IgG control group, the relative enrichment level of m^6^A on LINC01003 in glioma cells was significantly increased (Fig. 6A).

The online tool, m6A2Target, was used to explore the relationship between m^6^A enzymes with LINC01003. We found 4 “writer” enzymes (RBM15B, WTAP, METTL3, HAKAI) and 1 “eraser” enzyme (ALKBH5) that may perturb the expression of LINC01003 (Additional file 6: Fig. S3A). The GEPIA2 database showed that METTL3 had the highest positive correlation with LINC01003 in glioma tissues (Additional file 6: Fig. S3B–F). METTL3 (termed “writers”), the key component of the m^6^A methyltransferase complex, has been reported to regulate the malignant progression of glioma [27]. Previous research has shown that METTL3 siRNA treatment significantly knocked down the expression of METTL3 in U87MG and U251 cells [16]. We then examined the regulatory effect of METTL3 on LINC01003. In glioma cells, knockdown of METTL3 significantly reduced the expression of LINC01003 (Fig. 6B). An anti-m^6^A RIP assay combined with RT-qPCR indicated that the m^6^A modification of LINC01003 was remarkably decreased in LINC01003-silenced glioma cells (Fig. 6C). Collectively, these data suggested that METTL3-mediated m^6^A modification is associated with the upregulation of LINC01003 in glioma.

## Discussion

Glioma is the most common malignant tumor in the central nervous system [28]. Recent advances have found that numerous functional lncRNAs are abnormally expressed in glioma tissues. LncRNAs have been considered as central regulators in glioma progression [29]. A recent study demonstrated that LINC01003 acts as a suppressor in multiple myeloma [30]. In glioma, LINC01003 is highly expressed in glioma stem cells [5]. In this study, we identified a new lncRNA, LINC01003, which showed a significant trend of increased expression in glioma tissues. Importantly, we found that higher LINC01003 expression level is associated with poor prognosis in glioma patients. Furthermore, knockdown of LINC01003 significantly suppressed the proliferation of glioma cells in vitro and tumorigenesis in vivo. Based on these results, LINC01003 may represent a predictor of prognosis and potential therapeutic target for glioma therapy.

EMT is an important process that bestows cells an increased invasive or migrated phenotype. The main reason for the recurrence of glioma is largely attributed to the increased invasion and migration ability induced by EMT [31, 32]. Cadherins, including N-cad and E-cad, matrix metalloproteinase 2 (MMP2), and MMP9 were demonstrated to be EMT-associated proteins [33–35]. A loss-of-function assay indicated that LINC01003 can trigger the migration of glioma cells in vitro. This was confirmed by the observed upregulation of E-Cad and downregulation of N-Cad, MMP2, and MMP9. Our findings suggested that LINC01003 may be a crucial effector molecule that induces EMT in glioma.

To undercover the mechanism underlying LINC01003 in the tumorigenesis of glioma, RNA-seq analysis were performed. KEGG enrichment indicated that LINC01003 may be involved in a focal adhesion signaling pathway that is closely associated with cell proliferation and migration. Focal adhesion kinase (FAK), a central mediator of cell adhesion, was shown to play important roles in glioma cell proliferation and migration [36–38]. However, glioma-associated lncRNAs targeting focal adhesion signaling pathways have not been fully determined yet. In our study, we found LINC01003 knockdown repressed the phosphorylation of FAK. Interestingly, we identified a new signaling axis, LINC01003/CAV1/FAK, through which LINC01003 regulates focal adhesion in glioma. Notably, expression of CAV1 was significantly upregulated in high-grade glioma patients, and high expression of CAV1 is associated with a poor prognosis in glioma. Previous studies have demonstrated that CAV1 promotes glioma cell proliferation and vasculogenic mimicry and regulates focal adhesion by effecting FAK phosphorylation [26, 39]. Therefore, our study indicated that LINC01003 is a positive regulator in the CAV1/FAK signaling pathway in glioma cells.

To determine the upstream mechanism of LINC01003 overexpression in glioma, we investigated whether m^6^A methylation regulates LINC01003 expression. In our study, we combined a RIP assay with bioinformatics analysis and found that LINC01003 is significantly enriched with m^6^A methylation in glioma cells. The m^6^A methylation is the most common RNA modification in eukaryotic cells. Studies have demonstrated that m^6^A methylation exists in many lncRNAs and modulates almost all stages of the lncRNA life cycle, including transcription, nuclear export, splicing, and degradation. Therefore, the m^6^A methylation is related to cancer progression, including cell proliferation and migration [40–42]. However, the functions of m^6^A modification in lncRNAs remain largely unknown. METTL3 is a pivotal m^6^A methyltransferase and has recently been reported to be involved in lncRNA stability in tumor progression [43]. We found that METTL3 mediates the expression and m^6^A modification of LINC01003, suggesting that METTL3-mediated m^6^A modification may be responsible for the upregulation of LINC01003 in glioma.

## Conclusions

Our results demonstrated the pro-metastatic function of LINC01003 in the diffusion of glioma cells. Additionally, we uncovered a new signaling axis, LINC01003/CAV11/FAK, involved in the modulation of cell migration. Moreover, METTL3-mediated m^6^A modification may increase LINC01003 expression and function in glioma cells. Taken together, our present study provides a potential target that may serve as a promising prognostic biomarker and potential therapeutic target for glioma patients. Thus, clarifying the functions and mechanism of LINC01003 will provide anti-metastatic therapies in glioma.

## Supplementary Information

**Additional file 1**: **Table S1**. The DEGs between NC and LINC01003 smart silencer siRNA.

**Additional file 2**: **Table S2**. Top 20 KEGG enrichment pathways in the downregulated mRNAs.

**Additional file 3**: **Fig. S1**. CAV1 is upregulated in glioma tissues and cells.The expression of CAV1 in GBM, LGG, and normal brain tissues from the Gene Expression Profiling Interactive Analysis 2database.The expression of CAV1 in World Health Organizationgrade II–IV tumors from the Chinese Glioma Genome Atlasdatabase. Kaplan–Meier curves from theGEPIA2 orCGGA database determined the relevance between CAV1 expression and overall survival in each subtype or each grade of glioma patients.RT-qPCR was used to detect the expression level of CAV1 in U87MG and U251 cells with LINC01003 knockdown. * P < 0.05, ** P < 0.01, and ***P < 0.001.

**Additional file 4**: **Fig. S2**. The LINC01003/CAV1 axis regulates glioma in vitro and in vivo.A transwell assay was employed to determine the migration ability of U87MG cells. The migrating glioma cells were counted.Western blot was used to detect the expression levels of p-FAK in U87MG cells.An xenograft tumor model was generated by U87MG cells. The tumor volume and weight in nude mice were measured. Data represent the mean ± SD; * P < 0.05, ** P < 0.01, and ***P < 0.001.

**Additional file 5**: **Table S3**. The m6A sites on LINC01003 were predicted by the GEIPA2 database.

**Additional file 6**: **Fig. S3**. The relationship between m6A enzymes with LINC01003.m6A enzymes may perturb the expression of LINC01003.The correlation between m6A enzymes with LINC01003 in glioma tissues from the GEPIA2 database.

## Data Availability

The processed data that support the findings of this study are included in the manuscript and the supplementary materials.

## Abbreviations

CNS: Central nervous system
WHO: World Health Organization
lncRNAs: Long non-coding RNAs
ceRNAs: Competing endogenous RNAs
m^6^A: *N*^6^-Methyladenosine
UTRs: Untranslated regions
FAK: Focal adhesion kinase
GEPIA2: Gene expression profiling interactive analysis 2
CGGA: Chinese glioma genome atlas
HEB: Human astrocyte cell line
ATCC: American type culture collection
DMEM: Dulbecco's modified eagle medium
FBS: Fetal bovine serum
NC: Negative control
GBM: Glioblastoma multiforme
LGG: Lower grade glioma
EMT: Epithelial–mesenchymal transition
KD: Knockdown
KEGG: Kyoto encyclopedia of genes and genomes
DEGs: Differentially expressed genes
ECL: Enhanced Chemiluminescence
RIP: RNA imunoprecipitation
