# Supplementary figures and images for: m6A-mediated upregulation of LINC01003 regulates cell migration by targeting the CAV1/FAK signaling pathway in glioma
Source: Biol Direct. 2023 Jun 3;18:27. doi: 10.1186/s13062-023-00386-6 (PMC10239147; doi:10.1186/s13062-023-00386-6)

Figure S1

A.

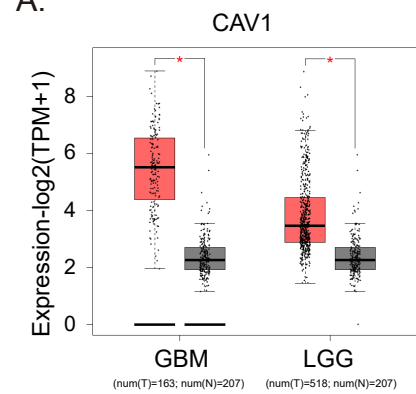

B.

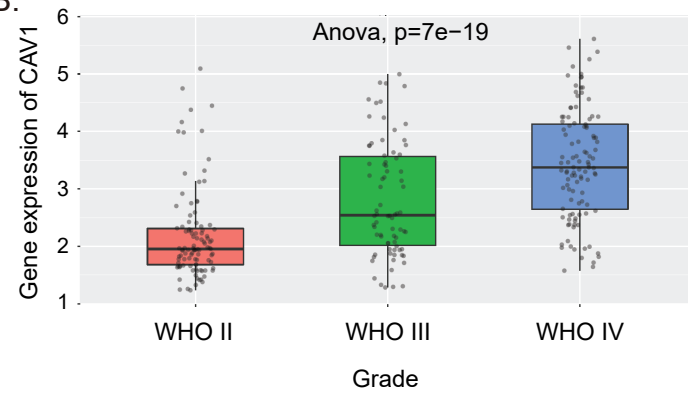

C.

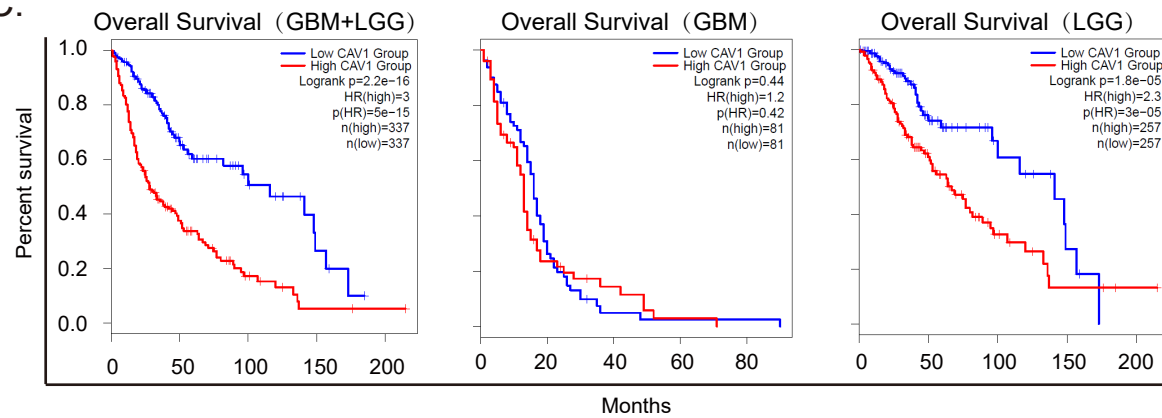

D.

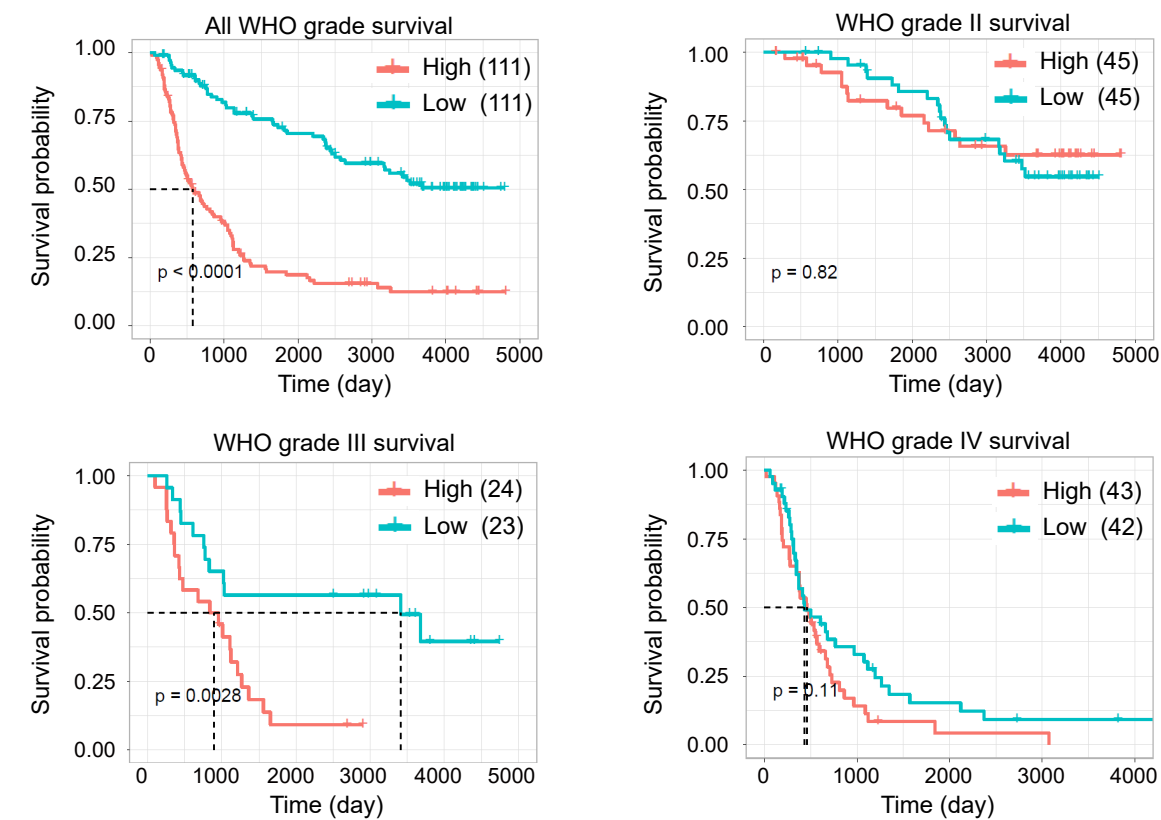

E.

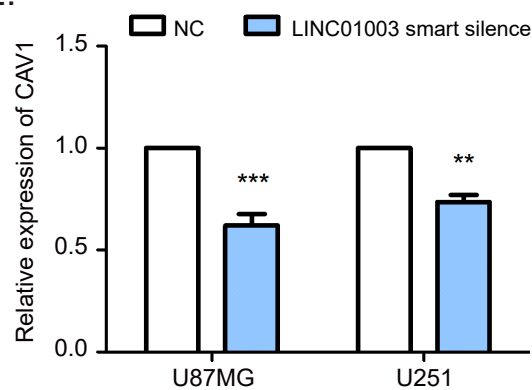

Supplement: Supplementary file 3 — Additional file 3: Fig. S1. CAV1 is upregulated in glioma tissues and cells.The expression of CAV1 in GBM, LGG, and normal brain tissues from the Gene Expression Profiling Interactive Analysis 2database.The expression of CAV1 in World Health Organizationgrade II–IV tumors from the Chinese Glioma Genome Atlasdatabase. Kaplan–Meier curves from theGEPIA2 orCGGA database determined the relevance between CAV1 expression and overall survival in each subtype or each grade of glioma patients.RT-qPCR was used to detect the expression level of CAV1 in U87MG and U251 cells with LINC01003 knockdown. * P < 0.05, ** P < 0.01, and ***P < 0.001. [file 13062_2023_386_MOESM3_ESM.pdf]

Figure.S2

A.

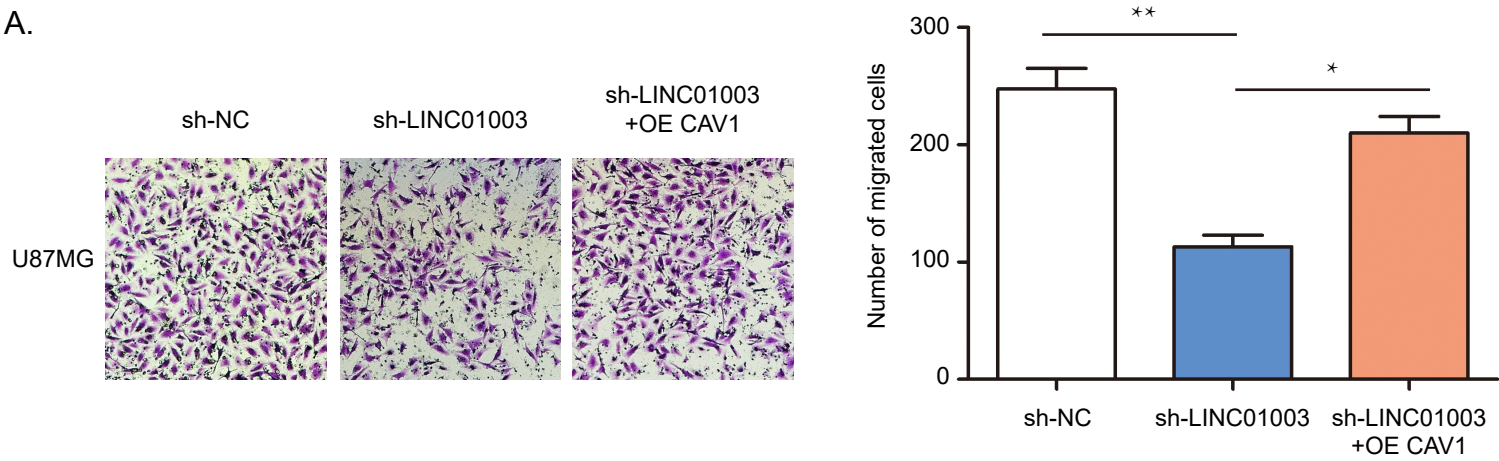

B.

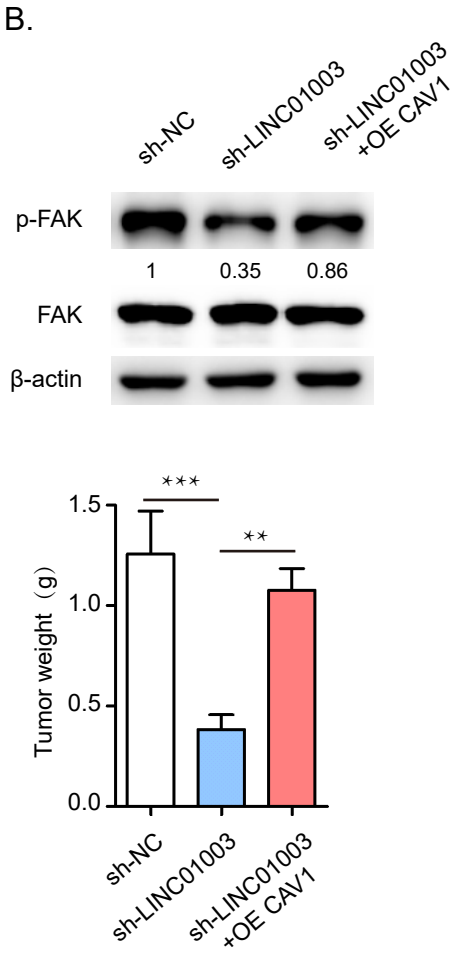

C.

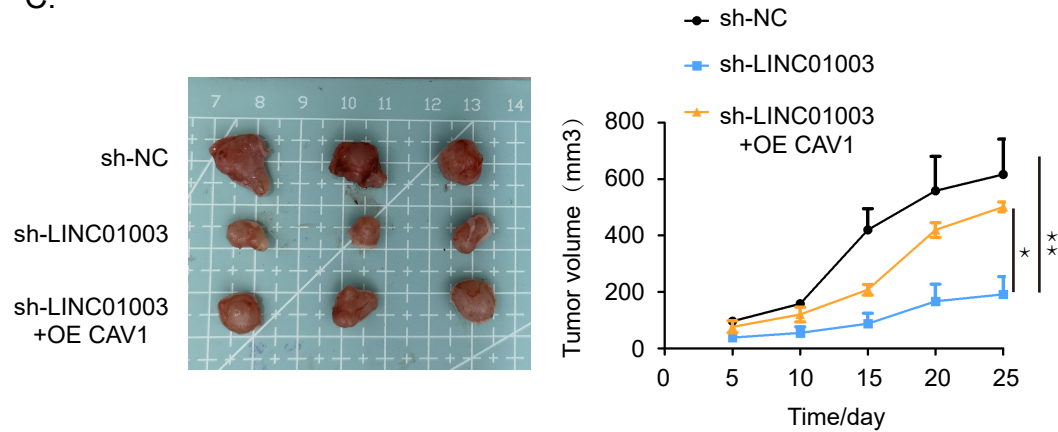

Supplement: Supplementary file 4 — Additional file 4: Fig. S2. The LINC01003/CAV1 axis regulates glioma in vitro and in vivo.A transwell assay was employed to determine the migration ability of U87MG cells. The migrating glioma cells were counted.Western blot was used to detect the expression levels of p-FAK in U87MG cells.An xenograft tumor model was generated by U87MG cells. The tumor volume and weight in nude mice were measured. Data represent the mean ± SD; * P < 0.05, ** P < 0.01, and ***P < 0.001. [file 13062_2023_386_MOESM4_ESM.pdf]
