# Supplementary material for: m6A-mediated upregulation of LINC01003 regulates cell migration by targeting the CAV1/FAK signaling pathway in glioma
Source: Biol Direct. 2023 Jun 3;18:27. doi: 10.1186/s13062-023-00386-6 (PMC10239147; doi:10.1186/s13062-023-00386-6)

Figure S3

A.

Name	Class	Cell line
RBM15B	Writer	HEK293T
WTAP	Writer	HEK293T
METTL3	Writer	HeLa, MOLM13, HepG2
HAKAI	Writer	HeLa
ALKBH5	Eraser	glioblastoma stem-like cell

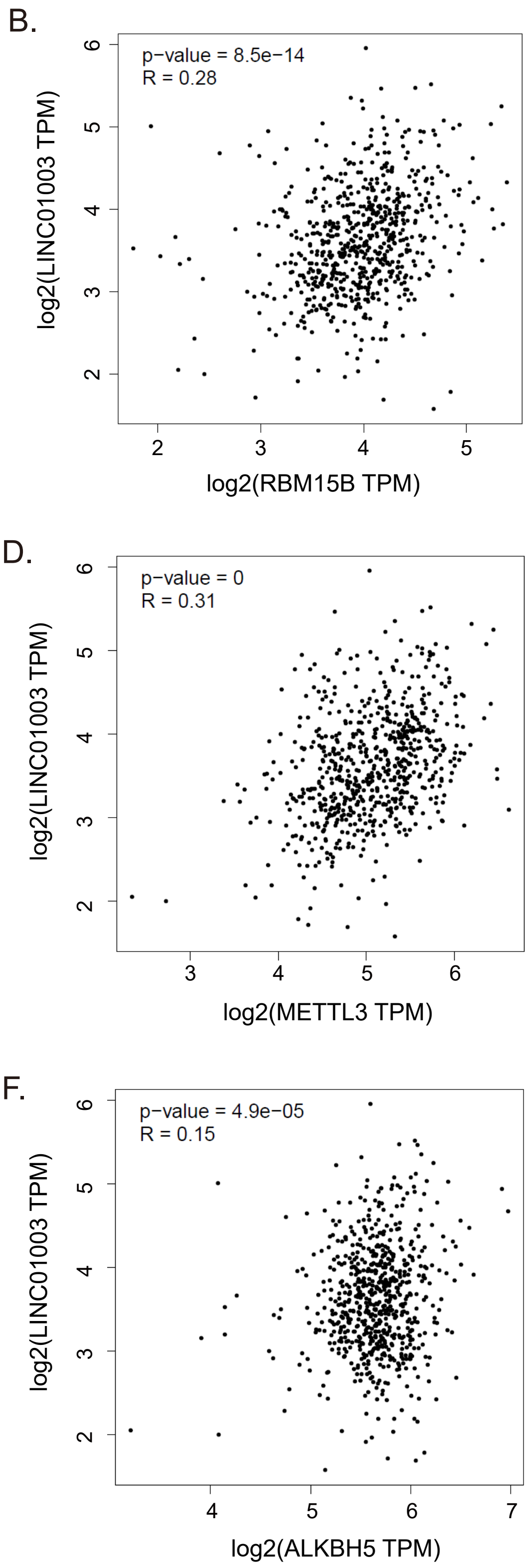

Supplement: Supplementary file 6 — Additional file 6: Fig. S3. The relationship between m6A enzymes with LINC01003.m6A enzymes may perturb the expression of LINC01003.The correlation between m6A enzymes with LINC01003 in glioma tissues from the GEPIA2 database. [file 13062_2023_386_MOESM6_ESM.pdf]
